# Care & custody: E-sport and patient-professional power-relations in forensic psychiatry. A qualitative study

**DOI:** 10.1192/j.eurpsy.2021.1011

**Published:** 2021-08-13

**Authors:** M. Terkildsen, H. Kennedy, A. Di Lieto, B. Jensen, L. Uhrskov

**Affiliations:** 1 Department Of Forensic Psychiatry, Aarhus University Hospital Psychiatry, Aarhus N, Denmark; 2 Defactum, Central Denmark Region, Aarhus N, Denmark; 3 Department Of Psychiatry, Trinity College- Dublin University, Dublin, Ireland; 4 National Forensic Mental Health Service, Central Mental Hospital Dundrum, Dundrum, Ireland; 5 Institute Of Clinical Medicine, Faculty of Health, Aarhus University, Aarhus, Denmark

**Keywords:** forensic psychiatry, qualitative study, care-custody, relations

## Abstract

**Introduction:**

Recovery orientated care emphasizes equality in relations. Forensic psychiatric professionals need to engage in care-relationships with patients in ways where power is symmetrically distributed among them. However, professionals also need to focus on security at the ward. This promotes patient-professional power-relations that are asymmetrically skewed towards professionals. New practical ways of balancing between the power-relations defined by a care and custody dichotomy in forensic care need to be developed and studied to guide clinical practice.

**Objectives:**

To study how power-relations are articulated between patient-professional within a social gaming activity (E – sport) in a Danish medium secure forensic psychiatric ward.

**Methods:**

Three months of observational data, collected via anthropological fieldwork Interviews with 3 professionals and 6 patients Data was analyzed using sociologist Pierre Bourdieu’s notions of field, capital and power

**Results:**

The E-sport intervention consists of two fields “in-game” and “over-game” In-game concerns the practice of gaming Over-game concerns the interventions organization Power in each field is driven by specific values and access to certain competencies Power in-game was equally open to patients and professionals leading to symmetric power relations Power over-game was open to professionals only leading to asymmetrical power relations Professionals may allow power distribution to patients during gameplay, while still retaining the overall power over the intervention

**Conclusions:**

It is possible to balance between care-and-custody in forensic psychiatry. This study provides important insights to guide further practice.

